# Monocyte trafficking to the brain with stress and inflammation: a novel axis of immune-to-brain communication that influences mood and behavior

**DOI:** 10.3389/fnins.2014.00447

**Published:** 2015-01-21

**Authors:** Eric S. Wohleb, Daniel B. McKim, John F. Sheridan, Jonathan P. Godbout

**Affiliations:** ^1^Department of Psychiatry, Yale University School of MedicineNew Haven, CT, USA; ^2^Division of Biosciences, The Ohio State University College of DentistryColumbus, OH, USA; ^3^Department of Neuroscience, The Ohio State University College of MedicineColumbus, OH, USA; ^4^Institute for Behavioral Medicine Research, The Ohio State University College of MedicineColumbus, OH, USA; ^5^Center for Brain and Spinal Cord Repair, The Ohio State University College of MedicineColumbus, OH, USA

**Keywords:** stress, neuroimmune, microglia, monocytes, macrophages, anxiety, depression, post-traumatic stress disorder

## Abstract

**HIGHLIGHTS**

- Psychological stress activates neuroendocrine pathways that alter immune responses.
- Stress-induced alterations in microglia phenotype and monocyte priming leads to aberrant peripheral and central inflammation.
- Elevated pro-inflammatory cytokine levels caused by microglia activation and recruitment of monocytes to the brain contribute to development and persistent anxiety-like behavior.
- Mechanisms that mediate interactions between microglia, endothelial cells, and macrophages and how these contribute to changes in behavior are discussed.
- Sensitization of microglia and re-distribution of primed monocytes are implicated in re-establishment of anxiety-like behavior.

Psychological stress causes physiological, immunological, and behavioral alterations in humans and rodents that can be maladaptive and negatively affect quality of life. Several lines of evidence indicate that psychological stress disrupts key functional interactions between the immune system and brain that ultimately affects mood and behavior. For example, activation of microglia, the resident innate immune cells of the brain, has been implicated as a key regulator of mood and behavior in the context of prolonged exposure to psychological stress. Emerging evidence implicates a novel neuroimmune circuit involving microglia activation and sympathetic outflow to the peripheral immune system that further reinforces stress-related behaviors by facilitating the recruitment of inflammatory monocytes to the brain. Evidence from various rodent models, including repeated social defeat (RSD), revealed that trafficking of monocytes to the brain promoted the establishment of anxiety-like behaviors following prolonged stress exposure. In addition, new evidence implicates monocyte trafficking from the spleen to the brain as key regulator of recurring anxiety following exposure to prolonged stress. The purpose of this review is to discuss mechanisms that cause stress-induced monocyte re-distribution in the brain and how dynamic interactions between microglia, endothelial cells, and brain macrophages lead to maladaptive behavioral responses.

## Introduction

Psychological stress in humans promotes development of mental health disturbances, including anxiety and depressive disorders (Kendler et al., 1999; McLaughlin et al., 2010; Gilman et al., 2013). Nonetheless the biological mechanisms connecting stress to mental health complications are not well-understood. Recent evidence indicates that inflammation and altered immune signaling significantly contribute to the etiology of many psychiatric symptoms and disorders (Evans et al., 2005), particularly in the context of chronic stress (Miller et al., 2009), depression (Raison et al., 2006), and anxiety (Pace and Heim, 2012). Indeed, chronic psychological stress in humans causes a “transcriptional fingerprint” on peripheral monocytes that is characterized by increased pro-inflammatory-related gene expression, particularly relating to the NF-κB transcriptional control pathway (Miller et al., 2008, 2014; Cole et al., 2011, 2012; Powell et al., 2013). This “transcriptional fingerprint” of stress on monocytes is linked to exaggerated pro-inflammatory responses following *ex vivo* immune stimulation and reduced anti-inflammatory responses initiated by glucocorticoid (GC)-mediated transcription (Miller et al., 2002; Rohleder et al., 2009; Cohen et al., 2012; Rohleder, 2012). Thus, chronic psychological stress substantially enhances the pro-inflammatory profile of peripheral monocytes.

These associations between stress and inflammation are relevant because immune activity potently regulates mood and behavior (Dantzer et al., 2008). For example, patients treated with inflammatory cytokines experience severe mood disturbances and have increased prevalence of depression (Udina et al., 2014). Moreover, peripheral innate immune stimulation increased self-reported anxiety symptoms (Reichenberg et al., 2001). These links between inflammation and behavior are well-established, particularly in the context of depression (Raison et al., 2006) and recurrent anxiety disorders (Pace and Heim, 2012). Thus, these studies show that the associations between stress and inflammation are critical for understanding the underlying neurobiology of stress-related mood disorders. Many of these stress-related immune phenomena are recapitulated in rodent models of stress including restraint stress, chronic variable stress, inescapable foot shock, and repeated social defeat (RSD). The objective of this review is to discuss novel data regarding brain-to-immune and immune-to-brain communication that function to regulate mood and behavior.

**Figure 1:**
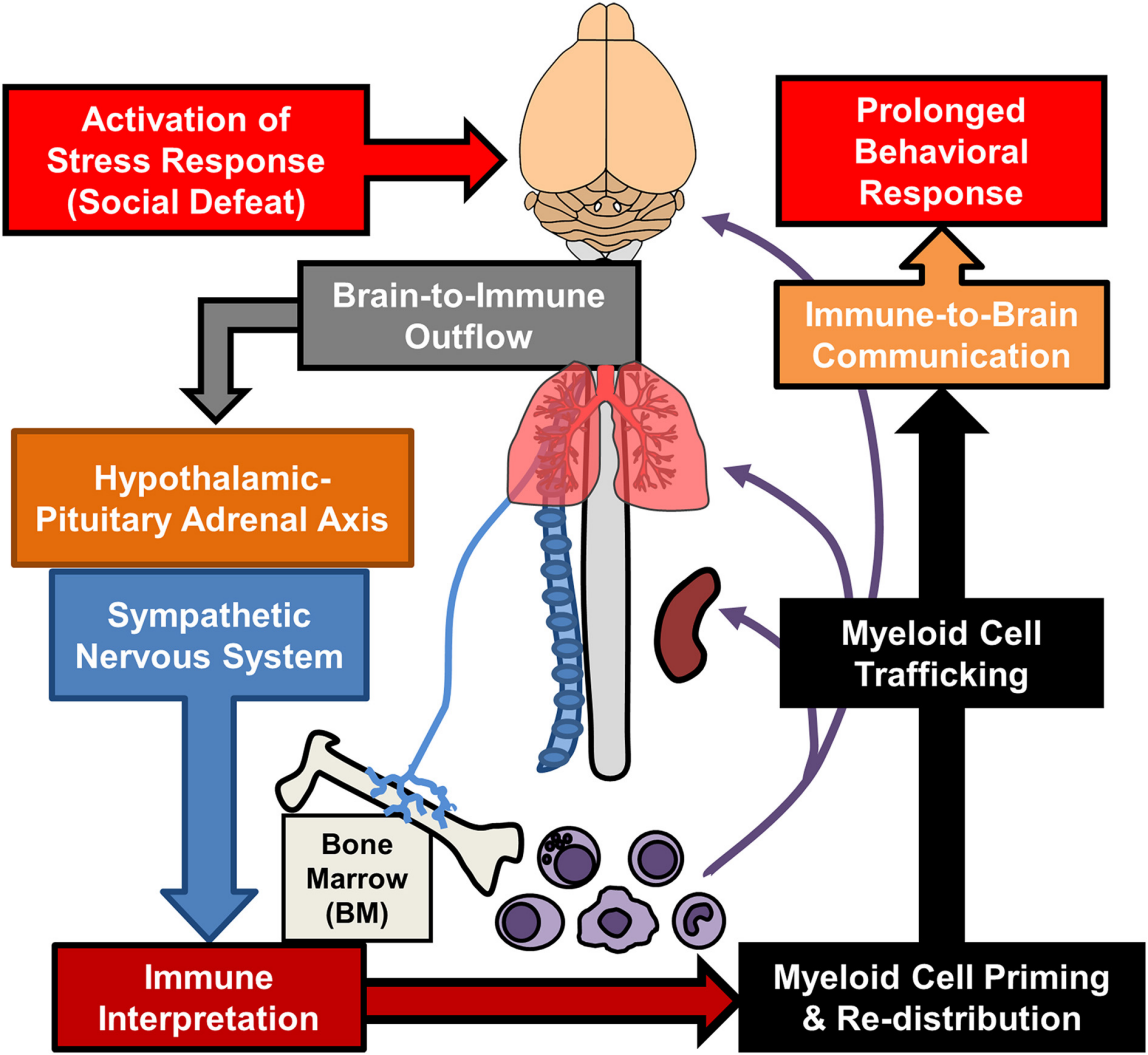
**Overview of bi-directional neuroimmune communication in response to stress**. Stress responses initiate brain-to-immune outflow that influences myeloid cell function. Re-distribution of primed myeloid cells augments immune-to-brain communication contributing to prolonged anxiety- and depressive-like behaviors.

Additional sections of this review will focus on recent findings from the RSD model that have elucidated new components of neuroimmune facilitation of stress-induced behavioral adaptations. One important concept is that RSD and other stressors are physiological in nature and are interpreted in the brain within discrete stress-responsive neurocircuitry. Activation of this neurocircuitry subsequently leads to activation of the sympathetic nervous system (SNS) and the hypothalamic pituitary adrenal axis (HPA). Activation of the SNS and HPA with stress allows the CNS to communicate with the immune system and to profoundly influence its functions (Sternberg, 2006). We will discuss mechanisms regarding how the immune system responds to prolonged stress and how immune modulation then feeds back to the brain to modulate mood and behavior. For example, as illustrated in Figure 1, RSD exposure causes SNS-dependent monocyte trafficking from the bone marrow (BM) to the brain. This leads to dynamic interactions between BM-derived monocytes, endothelial cells, and resident microglia. Together, these signals converge to augment neuroinflammatory signaling and promote prolonged anxiety. A final topic reviewed here is the recent evidence that a similar neuroimmune circuit contributes to recurring anxiety following RSD-induced stress-sensitization. These recent studies show that this neuroimmune circuit was re-activated following exposure to a secondary acute stress, and this resulted in the re-establishment of anxiety-like behavior that was dependent on monocyte re-distribution and microglia sensitization. Overall these novel findings indicate that psychological stress initiates a cascade of neuroimmune responses involving brain-to-immune and immune-to-brain signaling that converge to influence mood and behavior.

## Interpretation of stressful stimuli engages cortico-limbic brain regions and activates neuroendocrine responses

In both rodents and humans, physiological stress is interpreted by the brain within fear and threat appraisal circuitry that results in both neurobiological and behavioral responses. It is important to understand the cellular and molecular mechanisms mediating these behaviors because psychological stress in humans is associated with the development of anxiety, social withdrawal, and major depression (Ressler and Mayberg, 2007; Price and Drevets, 2010). Early studies examining the neurocircuitry of stress-responses in rodents showed that key stress-responsive areas of the brain are activated, including the pre-frontal cortex (PFC), hypothalamus (HYPO), amygdala (AMYG), and the CA3 and dentate gyrus of the hippocampus (HPC) (Kollack-Walker et al., 1997; Martinez et al., 2002). This is pertinent because there is evidence that activation of this neurocircuitry can manifest as anxiety or depression in humans (Sheehan et al., 2004). Thus, understanding fear and threat appraisal circuitry can help elucidate the neurobiology underlying the development of mood disturbances related to chronic stress.

**Figure 2:**
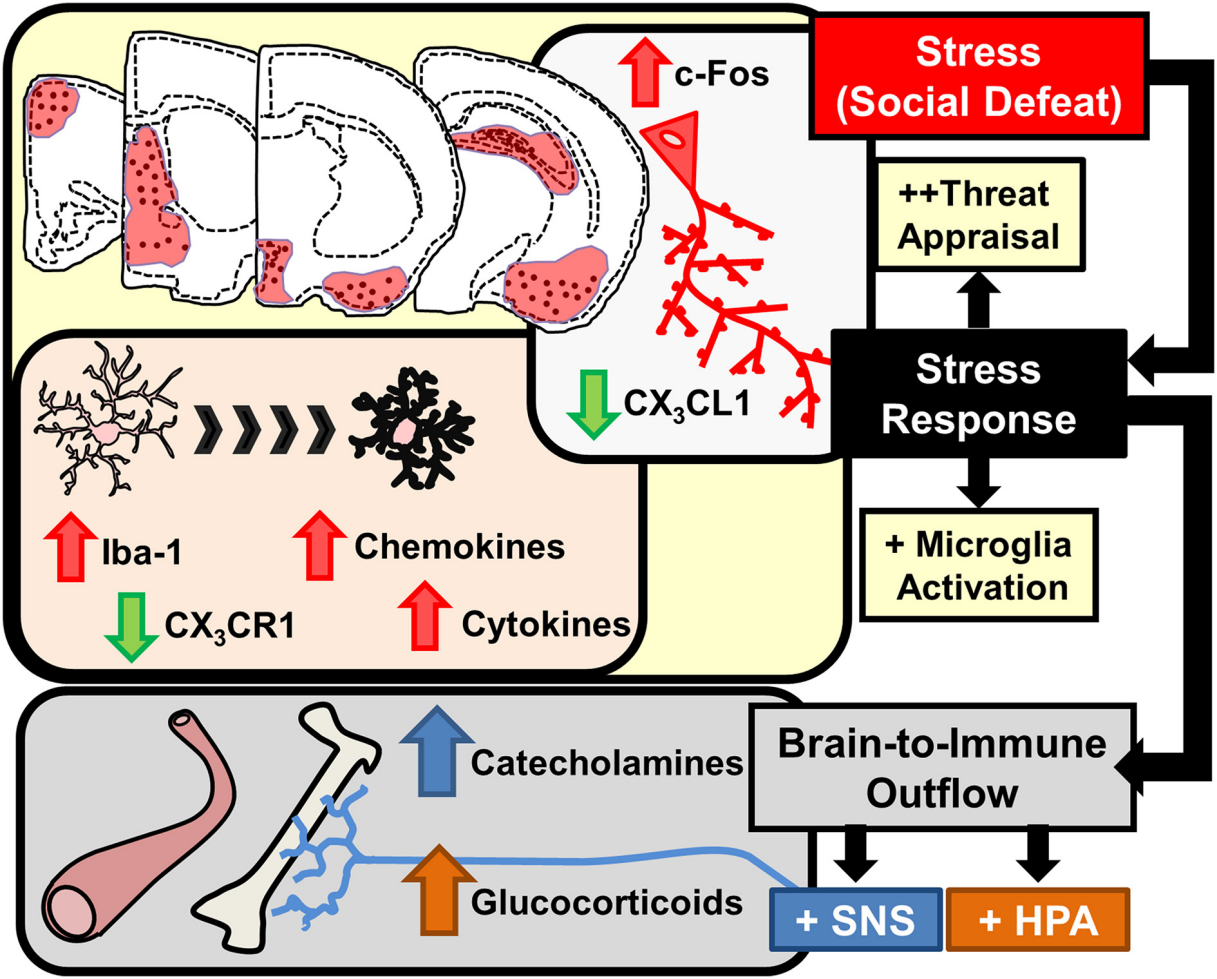
**Stress response in the brain leads to microglia activation and brain-to-immune outflow alters immune activity in peripheral immune organs**. Interpretation of stressful stimuli activates threat appraisal neurocircuitry including specific cortico-limbic brain regions that show increased c-Fos immunolabeling. In corresponding brain regions microglia demonstrate morphological changes, increased pro-inflammatory cytokine and chemokine expression and deficits in immunoregulatory markers such as CX_3_CR1 are evident. Stress responses also cause brain-to-immune outflow with HPA and SNS activation that profoundly alter peripheral immune activity.

Stress-responsive patterns of brain region activity are recapitulated in animal models of stress, such as RSD and restraint. This notion is supported by studies using c-Fos immunolabeling. c-Fos is an immediate early gene used as a functional marker of neuronal activation (Kovacs, 1998). For instance, social stress in rodents increased the number of cFos-expressing neurons in the PFC, HYPO, AMYG, and HPC (Kollack-Walker et al., 1997; Wohleb et al., 2011). In addition, several limbic brain regions implicated in regulating mood, including the bed nucleus of the stria terminalis (BNST), lateral septum (LS), and nucleus accumbens (NAc) are activated in response to social stressors (Martinez et al., 1998). While these forebrain and midbrain networks are interacting, visceral sensory information is simultaneously transmitted to cortico-limbic structures via brainstem nuclei that are innervated by several ascending pathways. For example, the vagus nerve communicates peripheral sensory events into these circuits via innervation of the nucleus of the solitary tract, which sends secondary projections to brainstem nuclei including the locus coereleus (LC). This is relevant because stress-induced autonomic activation can initiate arousal pathways originating in the LC. Projections from the LC release norepinephrine into distant regions, including the PFC, AMYG, and HPC, that cause enhanced attention and vigilance (Morilak et al., 2005; Radley et al., 2008; Samuels and Szabadi, 2008). Collectively these inter-connected brain regions constitute the key brain regions activated during stress exposure. Despite the fact that these brain regions appear to be detached, feedback mechanisms linking all of these structures together coordinate adaptive physiological and behavioral responses. Taken together, as illustrated in the top half of Figure 2, interpretation of stress involves neuronal activation within brain regions associated with fear and threat appraisal.

Several studies indicate that anxiety- and depressive-like behaviors are caused by stress-associated neurobiological alterations in brain regions, including the PFC, AMYG, and HPC (Ressler and Mayberg, 2007; Price and Drevets, 2010). For instance, stress exposure in rodents caused both retraction of neuronal dendrites and (Radley et al., 2004) and decreased dendritic spine density in the PFC (Magarinos et al., 1996). In addition, chronic stress impaired hippocampal neurogenesis (Gould et al., 1997, 1998). The hypothalamic-pituitary-adrenal (HPA) axis was implicated in these studies because exogenous glucocorticoid (GC) administration or blockade of endogenous stress-induced GCs resulted, respectively, in enhancement or reversal of these neurobiological effects. There are dichotomous findings in other regions such as the amygdala that show functional activation of the amygdala is enhanced in mood disorders (Sapolsky, 2003). Indeed, stress promoted neuronal hypertrophy and increased dendritic complexity in the amygdala of rodents (Vyas et al., 2002; Mitra et al., 2005). Neurobiological changes in the amygdala were also facilitated by hormonal release of GCs and central noradrenergic pathways (Roozendaal et al., 2009). Based on these data initial propositions suggested that stress hormones and neurotransmitters caused intrinsic neuronal adaptations leading to mood disorders (Krishnan and Nestler, 2008; Christoffel et al., 2011a,b).

Interpretation of psychological stress in the brain causes activation of neuroendocrine pathways that signal into the periphery, including the hypothalamic-pituitary-adrenal (HPA) axis and the SNS. HPA activation leads to release of glucocorticoids (GC) in circulation and activation of the SNS leads to increased release of catecholamines in circulation (epinephrine, Epi) and in tissues (norepinephrine, NE). Collectively, HPA and SNS activation with acute stress synergize to influence physiology with increased breakdown of glucose, increased heart rate and muscle tone (Sternberg, 2006). This response is termed the “fight or flight” response and provides the organism with increased energy availability and heightened awareness to respond to aversive challenges. Another major element of HPA and SNS activation is that these signals are relayed to the immune system (Irwin and Cole, 2011; Wohleb and Godbout, 2013). Thus, an integral component of the stress response is to relay information from the brain to peripheral organs and immune system via HPA and SNS neuroendocrine pathways (Figure 2).

## Stress-induced neuronal responses are associated with microglia activation and increased neuroinflammatory signaling

Integration of stress-induced signaling is facilitated in the brain by microglia, the resident immune cells of the brain, through propagation of neuroinflammatory signaling that modulates neuronal and endocrine responses to stress. Microglia are integral cellular components of the brain and partake in many homeostatic processes, including removal of apoptotic neurons, pruning of synapses, phagocytosis of excess proteins, and regulation of neurotransmitter levels (Tremblay et al., 2011). Microglia also have a role in immune surveillance, and when they become activated, they provide similar immune function as peripheral macrophages (Pocock and Kettenmann, 2007; Ransohoff and Perry, 2009; Kettenmann et al., 2011, 2013). This includes the production of inflammatory mediators, such as prostaglandins, cytokines, and chemokines. Based on their functional role in the brain, microglia are implicated in neuroinflammatory and behavioral responses to stress. For instance, numerous studies have revealed microglia activation and neuroinflammatory signaling occurs following prolonged stress exposure (Johnson et al., 2005; Frank et al., 2007a, 2012; Tynan et al., 2010; Wohleb et al., 2011, 2012, 2013; Bian et al., 2012; Hinwood et al., 2012, 2013; Kopp et al., 2013). Moreover, recent studies indicate that microglia activation and neuroinflammatory signaling have a causal role in behavioral responses to chronic stress (Blandino et al., 2006; Hinwood et al., 2012; Wohleb et al., 2013; Kreisel et al., 2014). In this context alterations in microglia physiology likely contribute to maladaptive neurobiological responses that underlie stress-induced mental health disorders. In this way, microglia may contribute to the maladaptive neurobiological interpretation of stress within the brain.

One common finding is that stress exposure altered microglia morphology in similar fear and threat appraisal areas of the brain that are activated by stress. For example, prolonged stress exposure caused neuronal activation and altered microglia morphology in overlapping stress-responsive regions, including the PFC, HYPO, AMYG, and the CA3 and dentate gyrus (DG) of the HPC (Tynan et al., 2010; Wohleb et al., 2011; Hinwood et al., 2013). In these studies, stress-induced morphological changes in microglia were consistent with an activated profile. In support of this idea, altered microglia morphology following RSD, foot shock, and chronic unpredictable stress were all associated with increased pro-inflammatory cytokine mRNA expression and exaggerated pro-inflammatory responses to immunological challenges (Frank et al., 2007a; Wohleb et al., 2011, 2012). In the context of psychological stress, the term “activated microglia” refers to changes in morphology (e.g., increased soma size) that corresponds with increased mRNA expression of inflammatory mediators including cytokines and chemokines. Thus, as highlighted in Figure 2, prolonged stress exposure caused microglia activation that regionally correlated with neuronal activation in stress-responsive areas of the brain.

The regional co-occurrence of neuronal and microglial activation suggests a causative link between these two events. In support of this, evidence from RSD revealed that increased region-specific neuronal activation was evident after a single cycle of social defeat. This neuronal activation preceded elevated cytokine expression that was observed only after at least 3 cycles. These findings are relevant because they indicated that neuronal activation is an upstream event of microglia activation. Moreover, pre-treatment with propranolol, a β-adrenergic receptor antagonist, blocked stress-induced neuronal activation as well as alterations in microglia morphology (Wohleb et al., 2011). Thus, β-adrenergic receptor-dependent neuronal activity was implicated in stress-induced microglia activation. Moreover, other reports revealed that central administration of β-adrenergic receptor agonists alone elicited pro-inflammatory cytokine production, and ablation of noradrenergic locus-coereleus (LC) projections reduced stress-induced IL-1β production in the HPC (Johnson et al., 2005). Furthermore, in a recent study, *in vitro* application of norepinephrine caused microglia morphological changes, including retraction of processes that was consistent with an activated phenotype (Gyoneva and Traynelis, 2013). These results indicate that central noradrenergic responses have a substantial contribution to stress-induced microglia activation and neuroinflammatory signaling.

The notion that neuronal activity regulates microglia activation is supported by the fact that microglia actively monitor neurons through CD200/CD200R interactions, chemokine signaling (i.e., CX_3_CL1), growth factors (i.e., M-CSF), ATP release, and neurotransmitter levels (Kettenmann et al., 2011; Kierdorf and Prinz, 2013; Dissing-Olesen et al., 2014). In the RSD model, region-specific microglia activation corresponded with reduced anti-inflammatory regulation through neuronal-derived fractalkine ligand (CX_3_CL1) and reduced fractalkine receptor (CX_3_CR1) on microglia (Figure 2). During physiological conditions CX_3_CL1 expression is highly enriched within neurons and its homeostatic expression promotes a less-inflammatory profile of microglia that ubiquitously express CX_3_CR1 (Cardona et al., 2006; Wynne et al., 2010). However, RSD caused significant reductions in CX_3_CL1 expression (Wohleb et al., 2013) that were compounded by decreased CX_3_CR1 expression in enriched microglia (Wohleb et al., 2014b). Moreover, the region specificity of microglia activation is also explained by the notion that neuronal CX_3_CL1 expression is reduced in an activity dependent manner (Harrison et al., 1998). Thus, region-specific neuronal activation following stress exposure corresponded with reduced CX_3_CL1 and CX_3_CR1 expression that may contribute to activation of microglia within stress-responsive areas of the brain.

In numerous models of stress, microglia are implicated as the source of neuroinflammatory signals. In these studies, minocycline, an antibiotic that limits microglia responses, prevented stress-induced pro-inflammatory cytokine expression in the brain. For example, minocycline prevented increased IL-1β expression in the brain following foot shock (Blandino et al., 2009), reduced swim stress-induced microglia NF-κB signaling (Bradesi et al., 2009), and attenuated restraint stress-induced microglial activation (Hinwood et al., 2012). A compelling observation with chronic restraint stress is that minocycline treatment also attenuated persistent neuronal activation, as indicated by reduced FosB neuronal labeling (Hinwood et al., 2012). This observation suggests that microglia activation may enhance or reinforce chronic stress-induced neuronal activity. Consistent with this, inhibition of microglia activation by minocycline or other anti-inflammatory interventions corresponded with attenuation of cognitive deficits, depressive-like behavior, and anxiety-like behavior following restraint stress (Hinwood et al., 2012) and chronic variable stress (Kreisel et al., 2014). Moreover, there is substantial evidence that microglia activation and brain cytokine signaling following stress-exposure augment neuroendocrine outflow that may further reinforce stress-related behaviors (Goshen et al., 2008). It is thought that microglia activation potentiates HPA responses via release of IL-1β within the hypothalamus (Goshen and Yirmiya, 2009). As illustrated in Figure 2, stress-induced microglia activation occurs within discrete stress-responsive brain regions, reinforces stress-responsive neuronal circuits, and augments neuroendocrine activation. This is important because these events influence immune and behavioral responses to stress.

## Neuroendocrine pathways signal to the immune system and increase the release of inflammatory myeloid cells from the bone marrow

As previously discussed, activation of the HPA axis and SNS relays stress interpretation from the brain to the immune system (bottom half of Figure 2). The best example of this communication is the hardwiring of the SNS into primary and secondary lymphoid tissues, including bone marrow (BM), lymph nodes, and spleen (Felten et al., 1985). In this context, stress-induced SNS activation causes direct release of catecholamines into these immune organs. This is pertinent because peripheral immune cells express receptors for NE, and stimulation of these receptors causes functional responses that influence their development, inflammatory phenotype, and migrational capacity (Bierhaus et al., 2003; Nance and Sanders, 2007; Grisanti et al., 2010). In the context of prolonged or repeated activation of the SNS, such as with chronic stress, increased NE in the BM promotes the production and release of myeloid cells, including monocytes and granulocytes (Dhabhar et al., 2012; Hanke et al., 2012). The increased cycling of myeloid cells in the BM with stress shifts the phenotype of peripheral monocytes to be less mature and more “inflammatory” (Engler et al., 2004, 2005; Hanke et al., 2012; Heidt et al., 2014). These monocytes are termed “inflammatory” because they are able to traffic throughout the body and have enhanced capacity to release pro-inflammatory cytokines upon entering tissue and becoming effector cells. This is relevant because stress-induced trafficking of inflammatory monocytes contributes to the exacerbation of both mental and physical health conditions (Dutta et al., 2012; Hanke et al., 2012; Liezmann et al., 2012; Seifert et al., 2012; Wohleb et al., 2013, 2014a; Heidt et al., 2014).

Numerous studies in mice and humans revealed that the SNS directly innervates the BM. The most salient evidence of this is the presence of tyrosine hydroxylase-expressing (TH^+^) axons observed throughout the BM (Afan et al., 1997; Nance and Sanders, 2007). Moreover studies revealed that this innervation regulates immune function under homeostatic conditions and during various challenges, such as inflammation and stress (Felten et al., 1985; Nance and Sanders, 2007). It is well-documented that various types of psychological stress enhance SNS signaling in the BM via dichotomous mechanisms depending on the duration of the stress. For example, acute stress enhances the turnover and release of NE within the BM (Tang et al., 1999; Hanke et al., 2012), while chronic stress causes structural alterations, characterized by increased innervation of TH^+^ axons that corresponds with enhanced sympathetic signaling (Heidt et al., 2014). These effects are also observed in other lymphoid tissues in which psychological stress caused re-organization and increased innervation by SNS inputs (Sloan et al., 2008). Thus, the SNS acts as an integral relay of stress-signals from the brain to the immune system.

Related to the effects of stress on SNS activity, stress duration also has dichotomous effects on BM functions. For example, acute and chronic stress-exposures have differential effects on the release, proliferation, and inflammatory capacity of progenitors in the BM that are related to the temporal dynamics of SNS signaling. With acute stress, SNS activation is associated with increased egress of leukocytes, especially myeloid cells (e.g., monocytes and granulocytes), from the BM that transiently accumulate in circulation (Engler et al., 2004; Dhabhar et al., 2012). Similar to acute stress, chronic stress maintains increased leukocyte release from the BM. However, the dichotomy of stress-duration is evident in the selective enhancement of myeloid but not lymphocyte proliferation in the BM. For example, chronic stress caused sustained monocyte and granulocyte egress from the BM that resulted in substantial accumulation of these cells in circulation (Powell et al., 2013; Heidt et al., 2014). These myeloid-enhancing effects of chronic stress are amplified by proliferation and expansion of myeloid progenitor cells in the BM that occurs concomitantly with reductions in lymphocytes and erythrocytes (Engler et al., 2004; Powell et al., 2013; Heidt et al., 2014). Moreover, this selective enhancement of myelopoiesis was dependent upon SNS activation. For example, β-adrenergic receptor blockade with propranolol or selective β_3_-receptor antagonism prevented stress-induced enhancement of myelopoiesis in both RSD and chronic variable stress models (Wohleb et al., 2011; Hanke et al., 2012; Heidt et al., 2014). Chronic variable stress, that is also often referred to as “chronic mild stress” and “chronic unpredictable stress,” is similar to RSD in that it repeatedly promotes increased neuroendocrine activation with reoccurring stress (Yalcin et al., 2005). Thus, enhanced production and release of monocytes and granulocytes in the BM was dependent upon SNS activity and resulted in substantial accumulation of these cells in circulation.

**Figure 3:**
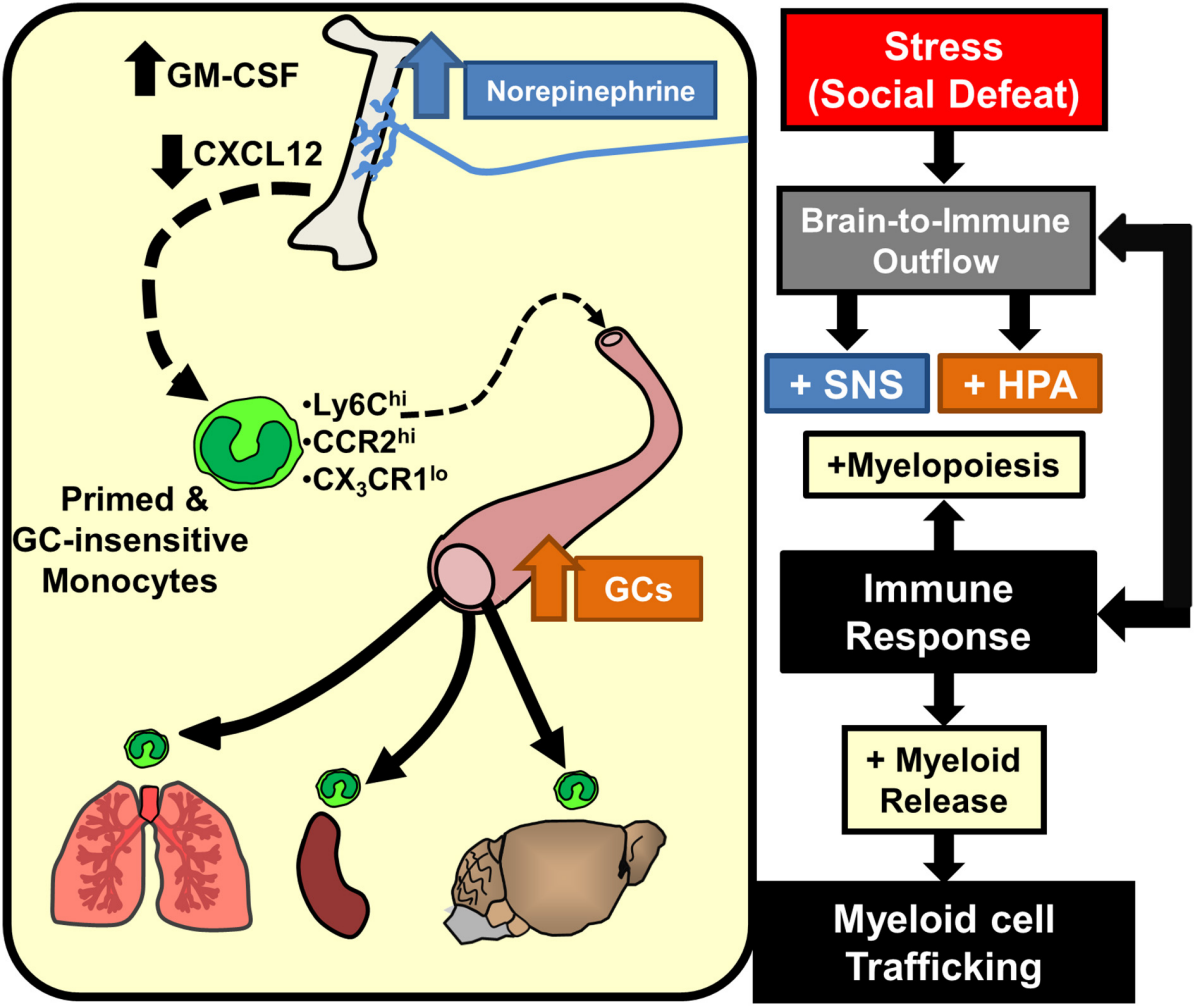
**Stress-induced brain-to-immune activation leads to enhanced myelopoiesis and monocyte trafficking**. SNS and HPA activation following stress exposure significantly shifts immune responses through increased myelopoiesis mediated by increased GM-CSF and reduced CXCL12 expression. Prolonged stress exposure promotes egress of primed and GC-insensitive monocytes (Ly6C^hi^/CCR2^hi^/CX_3_CR1^lo^) from the bone marrow into circulation. These monocytes have an increased capacity to traffic throughout the body and promote inflammation.

Studies in the RSD and chronic variable stress (CVS) models revealed key downstream signals provided by chemokines and growth factors that mediate SNS-dependent enhancement of myelopoiesis. With the CVS model, reduced CXCL12 signaling contributed to increased egress of myeloid cells from the BM (Heidt et al., 2014). This is consistent with the known function of CXCL12 to promote the retention of leukocytes (Katayama et al., 2006). Moreover, CXCL12 expression in BM-stromal cells was reduced by stress and rescued by β_3_-adrenergic blockade (Heidt et al., 2014). Thus, SNS-dependent reduction in stromal CXCL12 expression is a key regulator in the expansion of BM progenitors during chronic stress. Related to this, studies in the RSD model revealed that increased granulocyte-monocyte colony stimulating factor (GM-CSF) signaling mediates selective enhancement of myeloid expansion within the BM (Powell et al., 2013). It is relevant to note that myeloid cells share a common progenitor called a granulocyte-macropahge colony forming unit (GM-CFU) that also shares the common stimulatory growth factor, GM-CSF. Thus, increased expansion of granulocytes and monocytes in the BM following stress requires proliferation of GM-CFUs that is enhanced by GM-CSF signaling (Hamilton and Achuthan, 2013). In support of this notion, RSD increased BM GM-CSF expression in an exposure-dependent manner that temporally correlated with enhancement of myelopoiesis following 3 and 6 cycles of social defeat (Engler et al., 2005). Moreover, treatment with anti-GM-CSF antibody prevented enhanced myelopoiesis following RSD (Powell et al., 2013). Collectively, as illustrated in Figure 3, SNS stimulation of the BM results in reduced CXCL12 and increased GM-CSF that promotes the enhancement of myelopoiesis with prolonged stress exposure.

Activation of the SNS with prolonged stress not only regulates the production and release of myeloid cells but also enhances their pro-inflammatory profile. For example, numerous studies in humans reveal that chronic stress caused substantial enhancement of monocytic inflammatory potential. This is evidenced by exaggerated responses to *ex vivo* innate immune challenge (Miller et al., 2002; Rohleder et al., 2009; Cohen et al., 2012; Rohleder, 2012; Powell et al., 2013). The notion of stress-induced inflammation is best encapsulated in works published by Steve Cole, Gregory Miller, and colleagues, where they describe it as a “conserved transcriptional response to adversity” (Cole et al., 2011; Powell et al., 2013; Miller et al., 2014). These reports demonstrate that the “transcriptional fingerprint” associated with chronic stress is mainly characterized by up-regulation of pro-inflammatory transcription control pathways, particularly the NF-κB pathway (Miller et al., 2008, 2014; Cole et al., 2011, 2012). Moreover, chronic stress is associated with down-regulation of transcriptional activity mediated by the glucocorticoid (GC) receptor, resulting in functional glucocorticoid insensitivity and loss of anti-inflammatory feedback associated with GC signaling (Miller et al., 2002; Rohleder et al., 2009; Cohen et al., 2012; Rohleder, 2012). These SNS-mediated pro-inflammatory effects of stress are recapitulated in rodent models. For example, myeloid cells isolated from RSD-exposed mice exhibited a similar pro-inflammatory “transcriptional fingerprint” as humans exposed to chronic stress (Powell et al., 2013). Also similar to humans, RSD enhanced pro-inflammatory cytokine production following innate immune challenge (Avitsur et al., 2001, 2002, 2003; Stark et al., 2001, 2002; Quan et al., 2003; Bailey et al., 2004; Engler et al., 2005, 2008; Hanke et al., 2012). In these studies, increased cytokine production in response to immune challenge was associated with the development of GC-insensitivity in peripheral myeloid cells, in which they were resistant to apoptosis following treatment with high levels of GCs in *ex vivo* cultures. These data indicate that pro-inflammatory effects of chronic stress in humans are recapitulated with RSD.

A relatively unappreciated notion is that many of the pro-inflammatory effects of chronic stress are simply a function of enhanced myelopoiesis. For example, in both humans and rodents, enhanced myelopoiesis during prolonged stress results in selective accumulation of immature monocytes in the periphery (Engler et al., 2004; Wohleb et al., 2011; Heidt et al., 2014) that was directly linked to increased cycling and release of monocytes in the BM (Engler et al., 2004; Hanke et al., 2012; Heidt et al., 2014). The immature monocytes released during stress represent an inflammatory subset that are identified as Ly6C^hi^ in mice and as CD14^+^/CD16^−^ in humans (Geissmann et al., 2003; Heidt et al., 2014). They are considered immature because they are functional precursors of the matured and immunoregulatory Ly6C^lo^ or CD14^−^ subset (Murray and Wynn, 2011; Yona et al., 2013). Moreover, these cells are termed “pro-inflammatory” because they readily traffic to inflamed tissue and have robust capacity to secrete pro-inflammatory cytokines once they enter tissue and become effector cells (Serbina and Pamer, 2006). The immature nature of these monocytes is pertinent as it may also account for the development of GC-insensitivity following prolonged stress. For example, immature BM monocytes are functionally GC-insensitive (Fitting et al., 2004; Engler et al., 2005). Thus, accumulation of these innately GC-insensitive immature monocytes corresponds with the development of GC-insensitivity during chronic stress. In support of these points, blockade of stress-induced myelopoiesis by β-adrenergic antagonism prevented accumulation of Ly6C^hi^ monocytes (Hanke et al., 2012; Powell et al., 2013; Heidt et al., 2014), and this was associated with prevention of GC-insensitivity (Hanke et al., 2012) and reversal of pro-inflammatory transcriptional profiles following RSD (Powell et al., 2013). However, priming of splenic macrophages during RSD involves additional priming events associated with TLR ligation (Bailey et al., 2006, 2007, 2011). Taken together, SNS-dependent enhancement of myelopoiesis underlies peripheral inflammation following chronic stress via the accumulation of innately pro-inflammatory and GC-insensitive immature monocytes.

Another key characteristic of the pro-inflammatory monocyte phenotype associated with prolonged stress is their enhanced capacity to traffic and promote pro-inflammatory signaling throughout the body. For example, several studies using RSD demonstrated increased trafficking of Ly6C^hi^ monocytes to peripheral tissues that was associated with exaggerated cytokine responses (Wohleb et al., 2011, 2012; Hanke et al., 2012) and the exacerbation of inflammatory conditions (Bailey et al., 2009a,b; Curry et al., 2010; Dong-Newsom et al., 2010; Mays et al., 2010; Tarr et al., 2012). This is similar to work in the CVS paradigm, where prolonged stress increased trafficking of Ly6C^hi^ inflammatory monocytes that promoted inflammation and exacerbated pathology of vascular plaques of ApoE^−/−^ mice (Heidt et al., 2014). Thus, prolonged stress exposure substantially increased circulating Ly6C^hi^ monocytes that trafficked to tissue, promoted inflammation, and exacerbated pathology. It is important to note that peripheral inflammation and exacerbated pathology were reversible by the blockade of SNS-enhancement of myelopoiesis. For example, β_3_-adrenergic receptor antagonism attenuated monocyte trafficking and prevented the exacerbation of vascular plaques in ApoE^−/−^ mice exposed to CVS (Heidt et al., 2014). Similarly, propranolol prevented RSD-induced monocyte accumulation in BM, circulation, spleen, and brain that corresponded with reduced pro-inflammatory cytokine production in these tissues (Wohleb et al., 2011; Hanke et al., 2012). As summarized in Figure 3, increased trafficking of inflammatory monocytes following prolonged stress-exposure is due to SNS-mediated enhancement of myelopoiesis that results in the release of immature monocytes from the BM. These points are relevant, because it was recently demonstrated that stress causes these inflammatory monocytes to traffic and accumulate in the brain.

## Inflammatory monocytes released from the bone marrow traffic to the brain

It is well-described in models of trauma, neurological disease, or infection that inflammatory BM-derived monocytes traffic to and are recruited into inflamed tissue, including the brain and spinal cord (Donnelly and Popovich, 2008; Kigerl et al., 2009; McGavern and Kang, 2011). Recent evidence also indicates that there is significant trafficking and recruitment of peripherally derived monocytes to the brain with psychological stress (Brevet et al., 2010; Wohleb et al., 2011; Ataka et al., 2013; Wohleb et al., 2013, 2014a; Sawada et al., 2014). In these studies, monocytes traffic to the brain and differentiate into brain macrophages that promote inflammatory signaling. These reports are somewhat surprising because they indicate that monocyte trafficking to the brain occurs in the absence of overt tissue pathology or injury. Nonetheless trafficking of monocytes with stress is important because their accumulation in the brain can influence neuroinflammatory signaling and behavior (Terrando et al., 2011; Wohleb et al., 2011, 2013; Beumer et al., 2012; D'Mello et al., 2013; Degos et al., 2013; Sawada et al., 2014). In addition, increased presence of vascular-associated macrophages in the brain was recently implicated in depression. In this study, depressed individuals who committed suicide had increased perivascular Iba-1 immunolabeling compared to non-depressed controls (Torres-Platas et al., 2014). Thus, the trafficking of BM-derived monocytes to the brain with psychological stress represents a cellular pathway in which the immune system communicates back to the brain to regulate behavior.

BM-derived macrophages comprise an integral component of the innate immune system in the brain. These immuno-surveillant brain macrophages reside within the perivascular space, meninges, and choroid plexus, and represent a functionally distinct population in the brain separate from the resident microglia. These peripherally-derived macrophages (CD11b^+^/CD45^hi^/Iba-1^−/lo^) are steadily renewed every 3–4 weeks (Hickey and Kimura, 1988; Bechmann et al., 2001). Although brain macrophages comprise a small portion of resident immune cells, their proximity to vascular and parenchymal cells in the brain makes them an important transducer of peripheral immune signals to the brain (Serrats et al., 2010). Moreover, brain macrophages are significantly more efficient at antigen presentation and elicit more robust pro-inflammatory responses relative to microglia (Hickey and Kimura, 1988; Walker, 1999; Galea et al., 2007). The relative rarity of these brain macrophages is important because recent studies show that inflammatory conditions profoundly increased the presence of macrophages associated with the brain. Thus, it is plausible that accumulation of primed, GC-insensitive macrophages in the brain significantly influences neuroinflammatory signaling in response to stress.

In support of this idea, recent studies show that RSD increased the presence of peripherally derived macrophages in the brain. For example, RSD caused a two to three fold increase in the number of CD11b^+^/CD45^hi^ cells in the brain (Wohleb et al., 2011, 2013, 2014a), which were not resident microglia (Sedgwick et al., 1991; Ford et al., 1995). Additionally, these cells were Ly6C^hi^ and had scatter properties consistent with trafficking monocyte/macrophages (Wohleb et al., 2012). Based on these profiles, these cells were referred to as brain macrophages. The RSD-induced increases in brain macrophages persisted after perfusion of the vasculature (Wohleb et al., 2012) and occurred despite no disruption in the blood-brain barrier (BBB) (Wohleb et al., 2013). In addition, studies using transgenic LysM-GFP^+^ mice confirmed that CD11b^+^/CD45^hi^ brain macrophages were derived from peripheral myeloid cells. Notably, LysM is expressed on monocytes and macrophages but not on resident microglia (Faust et al., 2000; Kim et al., 2009). Further histological analysis revealed that RSD increased rod/circular LysM-GFP^+^ macrophages within the perivascular regions of the brain (Wohleb et al., 2013). While these LysM-GFP^+^ macrophages were apparent around the vasculature, LysM expression appeared to be down-regulated as the cells reached the parenchyma of the brain. Thus, parenchymal infiltration could not be assessed using these mice. Taken together, initial studies using flow cytometric markers and LysM-GFP expression revealed that RSD increased macrophages in the brain.

**Figure 4:**
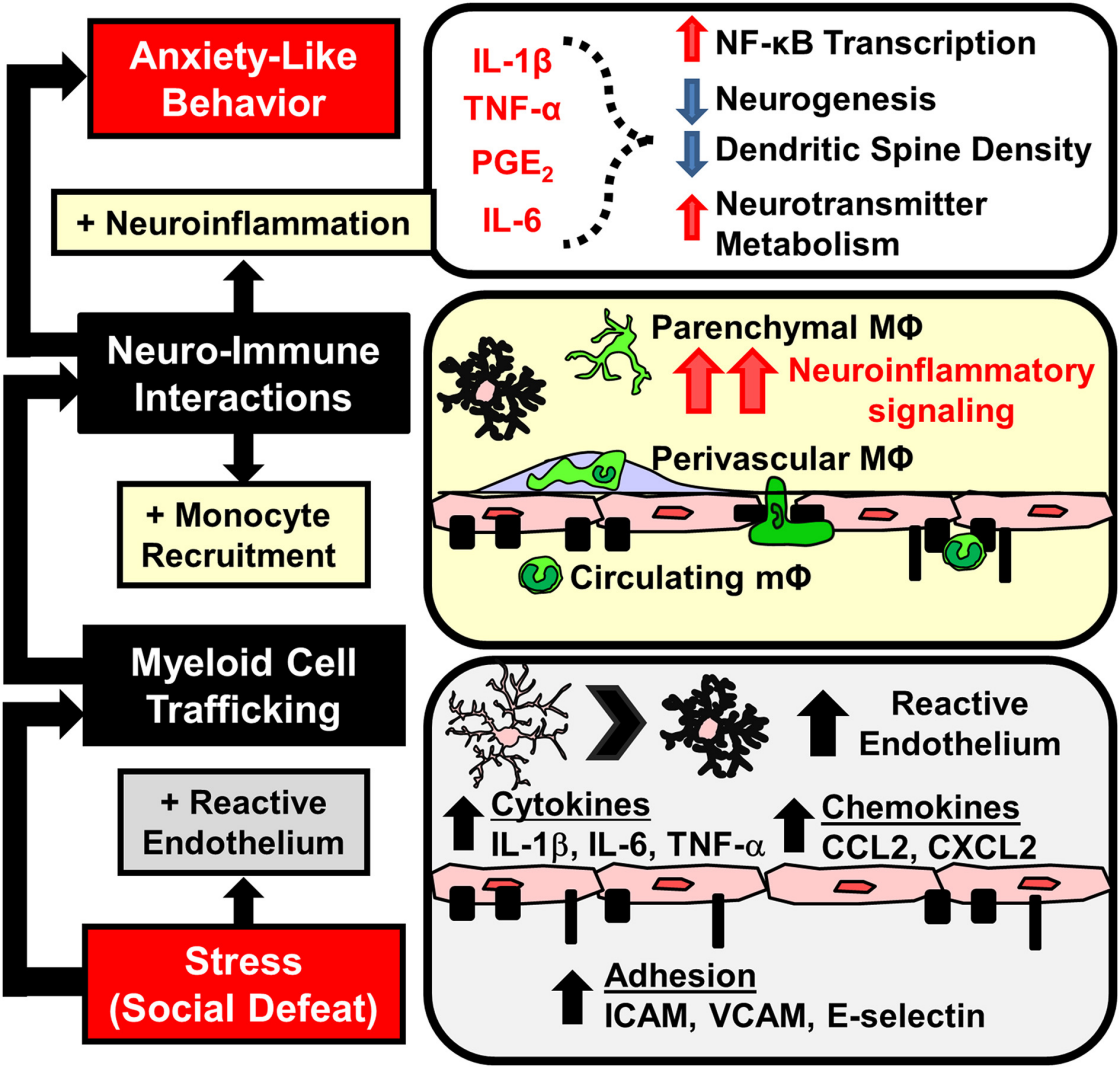
**Stress-induced microglia activation and macrophage recruitment to the brain contributes to development of prolonged anxiety-like behavior**. Repeated social defeat leads to microglia activation with increased pro-inflammatory cytokine and chemokine production that contributes to the development of reactive endothelium. Vascular endothelial cells increase cell adhesion molecule (CAM) expression that facilitates the adherence and extravasation of peripehrally derived monocytes (mΦ) that differentiate into perivascular and parenchymal macrophages (MΦ). Accumulation of MΦ in the brain converge with activated microlgia and amplify neuroinflammatory signaling. Downstream elevations in neuroinflammatory mediators (IL-1β, TNF-α, IL-6, prostaglandins) are implicated in neurobiological changes that promote anxiety-like behavior.

BM-chimera mice were used to further enable characterization of BM-derived macrophages in the brain. In these studies, host BM was ablated using low doses of busulfan and was then reconstituted with donor BM from mice that expressed green fluorescent protein (GFP) under a ubiquitous promoter. Thus, in these BM chimera mice, BM-derived immune cells would express GFP, while host-derived microglia would lack GFP expression. It is important to note that the low dose busulfan regimen used in these studies was not associated with trafficking of cells into the brain of control mice (Kierdorf et al., 2013; Wohleb et al., 2013). These studies confirmed the increased presence of BM-derived myeloid cells (GFP^+^/CD11b^+^) in the brain after RSD. The GFP^+^/CD11b^+^ cells in the brain after RSD were both CD45^hi^ and CD45^lo^ consistent, respectively, with perivascular and parenchymal localization (Mildner et al., 2007). Indeed, immunohistochemical studies detected GFP^+^ macrophages in both parenchymal and perivascular compartments of the brain. GFP^+^ macrophages persisted in the brain for at least 8 days after the cessation of RSD and were no longer present 24 days later (Wohleb et al., 2013, 2014a). Thus, studies using WT, LysM-GFP^+^, and BM chimera mice all detected macrophage accumulation in the brain following RSD. Overall, these data were interpreted to indicate that RSD increased the trafficking of BM-derived monocytes to the brain that differentiated into perivascular and parenchymal macrophages (Figure 4).

An important aspect of these studies with RSD was the selective infiltration of BM-derived monocytes into the brain parenchyma of stress-responsive regions (Wohleb et al., 2013). Notably, GFP^+^ macrophages accumulated in the same threat appraisal areas that had increased c-Fos and Iba-1 activation after RSD (Wohleb et al., 2011). Increased ramified GFP^+^ cells were specifically observed in brain regions associated with fear, anxiety, and threat appraisal, including the PFC, HYPO, AMYG, and CA3 and DG of the HPC, but were not observed in other brain regions like the motor cortex, striatum, somatosensory cortex, or cerebellum (Wohleb et al., 2011, 2013). Moreover, monocytes that infiltrated the parenchyma differentiated into ramified and Iba-1^+^ macrophages. In addition, infiltrating cells reduced expression of typical macrophage markers (e.g., CCR2, Ly6C, CD45, and LysM). Similar to prior reports, infiltrating macrophages had a microglia-like appearance, but had a less ramified morphology compared to resident microglia (Varvel et al., 2012; Elmore et al., 2014). Another point of interest was that similar to microglia activation (Wohleb et al., 2011), increases in parenchymal GFP^+^ macrophages were not observed until after 6 cycles of RSD (Wohleb et al., 2013). Thus, there was an exposure-dependent increase in brain macrophages after RSD. Overall, in the absence of BBB permeability, RSD caused region-specific infiltration of monocytes into brain regions associated with fear, anxiety, and threat appraisal.

The trafficking and recruitment of these monocytes with stress were also dependent on key chemokine receptors. For example, Wohleb et al. (2013) showed that CCR2^KO^ and CX_3_CR1^KO^ mice did not exhibit increased brain macrophages following RSD. Notably, RSD caused the release of myeloid cells into circulation independent of CCR2 or CX_3_CR1 expression, but neither CCR2^KO^ nor CX_3_CR1^KO^ mice had increased brain macrophages after RSD. These results indicate that stress-associated monocyte release alone was insufficient to gain access to the brain. In support of these findings, wild-type BM-chimera mice reconstituted with CCR2^KO^ or CX_3_CR1^KO^ progenitor cells also showed reduced brain macrophage trafficking following RSD. In addition, RSD increased CCL2 expression in the brain (Wohleb et al., 2013). These data indicate that monocytes were actively recruited to the brain after RSD using dynamic chemokine receptor interactions. The necessity for both CCR2 and CX_3_CR1 is supported in other models. For instance, infiltrating monocytes expressed CCR2 and CX_3_CR1 in the brain of experimental autoimmune encephalomyelitis and herpes-infected mice (Saederup et al., 2010; Boivin et al., 2012). Taken together, recruitment of monocytes to the brain with stress was prevented by deletion of key chemokine receptors, CCR2 and CX_3_CR1.

The notion that psychological stress increased the presence of BM-derived macrophages in the brain is supported by other studies. For example, BM-derived macrophages were detected in the ventral HPC in mice after five days of footshock stress (Brevet et al., 2010). These studies used a GFP^+^ BM-chimera generated by radiation in which the head was shielded. Consistent with studies using RSD, GFP^+^ cells showed a de-ramified, microglia-like morphological phenotype (Brevet et al., 2010). A similar set of studies in mice showed that simply observing cage mates undergoing foot shock was sufficient to cause influx of peripherally derived macrophages into brain parenchyma (Ataka et al., 2013). In these studies, both CCR2 and β-adrenergic receptor signaling contributed to the trafficking of BM-derived monocytes to the brain (Brevet et al., 2010; Ataka et al., 2013). In a more recent chronic neuropathic pain study, partial sciatic nerve ligation induced infiltration of BM-derived monocytes into the AMYG (Sawada et al., 2014), and this was blocked by oral administration of either a CCR2 antagonist or microinjections of IL-1 receptor antagonist. Thus, there is evidence for stress-induced brain-macrophage trafficking with several stress paradigms.

A relevant point to highlight is that infiltrating BM-derived monocytes/macrophages may not be identifiable with conventional lineage-specific markers (i.e., CD45, Ly6C, CD163, CCR2, LysM). This is because micro-environmental cues from the brain parenchyma cause macrophages to resemble microglia in both morphology and marker expression (Mildner et al., 2007). Numerous studies have reported that stress increased Iba-1^+^ cell numbers in stress-responsive brain regions (Frank et al., 2007b; Tynan et al., 2010; Bian et al., 2012; Kreisel et al., 2014). These data are often interpreted as microglia proliferation, but the increase in Iba-1^+^ cells may also be caused by influx of BM-derived macrophages in the brain. Thus, it is important to distinguish the role of resident microglia and infiltrating monocytes when studying stress and neuroinflammatory signaling. Collectively, several lines of evidence reveal that stress causes the recruitment and accumulation of BM-derived macrophages in the brain that involve chemokine signaling.

## Neurovascular dynamics facilitate stress-induced monocyte trafficking

Previous studies demonstrate that exposure to psychological stress elicited monocyte trafficking to the brain (Brevet et al., 2010; Wohleb et al., 2011, 2013, 2014a,b; Ataka et al., 2013; Sawada et al., 2014). It was discussed how neuroendocrine outflow from the brain to the immune system promotes the release of myeloid cells from the BM into circulation (Hanke et al., 2012; Heidt et al., 2014) (Figure 3). However, the release of inflammatory monocytes from the BM is insufficient to cause their active recruitment to the brain. For example, recruitment of monocytes to the brain following stress required chemokine signaling involving CX_3_CR1 and CCR2 (Ataka et al., 2013; Wohleb et al., 2013; Sawada et al., 2014). Moreover, accumulation of macrophages in the brain occurs with chronic peripheral inflammation that is also regulated by similar chemokine receptor and adhesion molecule dynamics (Kerfoot et al., 2006; D'Mello et al., 2009, 2013; Terrando et al., 2011; Degos et al., 2013). Thus, in the absence of frank neuropathology, the recruitment of monocytes into perivascular and parenchymal regions of the brain likely involves dynamic interactions between cells of the neurovascular unit, including endothelial cells, microglia, and neurons (Figure 4). The notion of a neurovascular unit is used to describe micro-dynamic interactions between the vasculature and local parenchymal cells, such as microglia and neurons (Stanimirovic and Friedman, 2012). This notion is appreciated in models of neurological diseases with inflammatory components, where neurovascular cells facilitate macrophage trafficking that can regulate disease progression (Prinz and Priller, 2010, 2014; Stanimirovic and Friedman, 2012). Here it is important to note that RSD promotes microglia activation and neuroinflammatory signaling that is independent of monocyte-trafficking. For example, deletion of CCR2 and CX_3_CR1 prevented stress-induced monocyte trafficking to the brain, but it did not prevent increased cytokine mRNA expression following RSD (Wohleb et al., 2013). It is also important to note that macrophage influx was observed within the same stress responsive regions where RSD caused increased neuronal c-Fos expression and altered microglia morphology (Wohleb et al., 2011, 2013). Related to this, recent evidence indicates that expression of key immune cell adhesion molecules (CAMs) occurs in specific brain regions after stress. For instance, ICAM-1 and VCAM-1, which are integral to monocyte-vasculature adhesion, were both increased on endothelial cells in the PFC and PVN following RSD (Sawicki et al., 2014). Similar to the patterns of macrophage trafficking (Wohleb et al., 2013), increased ICAM-1 and VCAM-1 regionally correlated with previous reports of neuronal and microglia activation within specific stress-responsive brain regions. These findings are consistent with the notion that neuronal-microglial-endothelial cross talk results in regions specific facilitation of macrophage trafficking via neurovascular CAM and chemokine expression (Figure 4). These dynamics are important because the presence of these monocytes/macrophages is implicated in pathogenesis of mental health disorders (Beumer et al., 2012; Torres-Platas et al., 2014). Thus, a key question is: to what degree does influx of monocytes influence neurocircuitry and behavior?

## Monocyte trafficking to the brain influences behavior

Several clinical and pre-clinical studies indicate that stress promotes the onset of anxiety- and depressive-like behaviors (Kendler et al., 1999; McLaughlin et al., 2010; Gilman et al., 2013). In the RSD model, anxiety-like behavior in the open field and light-dark preference tests developed after RSD (3–6 cycles) and persisted for at least 8 days (Wohleb et al., 2013, 2014a). It is important to note that behavioral responses to RSD are determined hours, days, and weeks after the cessation of the stressor. Thus, the measured behaviors are uncoupled from the transient induction of neuronal and endocrine arousal responses (Kinsey et al., 2007; Hanke et al., 2012). As previously outlined, studies with RSD indicate that inflammatory monocytes are released into circulation and they traffic to the brain. In this section, we will discuss the evidence that myeloid trafficking to the brain is necessary for the development of prolonged anxiety-like behavior following RSD (Figure 4). This section will review additional reports from other models that monocyte-trafficking is an important regulator of behavior.

With RSD, there are temporal relationships between the development, resolution, and recurrence of stress-induced anxiety and neuroimmune signaling. For example, anxiety, pro-inflammatory cytokine production in the brain, and brain macrophage accumulation were all observed in an exposure dependent manner after RSD. All parameters were moderately increased after 3 cycles and peaked after 6 cycles of social defeat (Wohleb et al., 2013). Similar to this, the resolution of anxiety and brain monocyte trafficking was also correlated. For instance, both anxiety-like behavior and increased brain macrophages persisted for at least 8 days after RSD and both of these parameters were resolved by 24 days later (Wohleb et al., 2014a). It should be noted, however, that social avoidance to an aggressor mouse developed after 1 cycle of social defeat and was maintained 24 days later (Wohleb et al., 2014a). Based on the timing and the lack of macrophages in the brain at these early and late time points, social avoidance occurs independent of monocyte trafficking to the brain. Overall, the development, maintenance, and resolution of anxiety, neuroinflammatory signaling, and brain monocyte trafficking in RSD were temporally correlated.

A key aspect to determining the cause and effect relationship between anxiety-like behavior and brain-monocyte trafficking was experimental interventions that prevented monocytes from trafficking to the brain. For example, initial studies used interventions that prevented release of monocytes from the BM (Engler et al., 2008; Wohleb et al., 2011), and this corresponded with blockade of both anxiety-like behavior and monocyte trafficking to the brain. For example, pretreatment with propranolol prevented the release and trafficking of monocytes from the BM to the brain (Wohleb et al., 2011), and this was associated with the absence of the stress-induced anxiety-like behavior in treated mice. Moreover, IL-1 receptor type-1 (IL-1R1)-knockout mice do not exhibit stress-induced brain-monocyte trafficking, and this was also associated with the absence of anxiety-like behavior (Wohleb et al., 2011, 2014b). These initial data were circumstantial in that they reveal strong correspondence between trafficking of monocytes to the brain and the development of anxiety-like behavior. In addition, subsequent studies revealed compelling evidence that direct interference of monocyte trafficking to the brain prevented anxiety-like behavior in RSD exposed mice.

To specifically interfere with monocyte trafficking without affecting stress-interpretation or monocyte priming, Wohleb et al. (2013) used transgenic mice deficient in key monocyte chemokine receptors. This study showed that both CCR2 and CX_3_CR1 expression were required for RSD-induced trafficking of monocytes to the brain. For example, both CCR2^KO^ mice and CX_3_CR1^KO^ mice had stress-induced changes in the circulating Ly6C^hi^ monocytes but neither had increased macrophages in the brain (Wohleb et al., 2013). It is important to note that chemokine deficiency was associated with blockade of stress-induced accumulation of both CD45^hi^ perivascular macrophages and GFP^+^ parenchymal macrophages, as studied in both naïve and BM-chimeric mice (Wohleb et al., 2013). In these examples, when macrophages were not able to reach the brain, mice did not exhibit anxiety-like behavior 14 h after RSD. Notably, prevention of brain-monocyte trafficking in knock-out mice was not associated with attenuation of increased IL-1β mRNA expression. These results suggest that intrinsic neuroinflammatory signaling is not sufficient to promote extended anxiety-like responses. Thus, the initial interpretation of these data is that brain-monocyte trafficking synergistically promotes the development of prolonged anxiety like-behavior following RSD.

A more generalized interpretation of these data is that trafficking of inflammatory monocytes to the brain is an independent axis of immune-to-brain signaling that regulates mood and behavior. In fact, there is additional evidence from other models to support this interpretation. For example, in a model of neuropathic pain, trafficking of macrophages to the amygdala promoted the development of anxiety-like behavior (Sawada et al., 2014). In this study, blockade of macrophage influx in the amygdala by CCR2 and IL-1R1 inhibition prevented pain-induced anxiety-like behavior. Additionally, the notion that monocyte trafficking to the brain influences behaviors has been reported in several models of peripheral inflammation. For example, experimental liver inflammation and experimental colitis both promote monocyte trafficking to the brain that is dependent upon CCL2/CCR2 signaling, tumor necrosis factor receptor expression, and P-selectin (Kerfoot et al., 2006; D'Mello et al., 2009, 2013). In these studies, blockade of brain-monocyte trafficking prevented inflammation-induced sickness behaviors. Moreover, brain monocyte trafficking promoted the development of cognitive decline following post-operative recovery from peripheral surgery (Terrando et al., 2011; Degos et al., 2013). These studies showed that general surgery caused accumulation of ramified CCR2^+^ macrophages in the hippocampus. Macrophage accumulation was prevented by depletion of peripheral phagocytes by injection of clodronate-loaded liposomes (Degos et al., 2013) and by inhibition of peripheral macrophage NFκB signaling (Terrando et al., 2011). Prevention of macrophage accumulation corresponded with prevention of postoperative cognitive decline (Terrando et al., 2011; Degos et al., 2013). Taken together, there is mounting evidence that trafficking of inflammatory monocytes to the brain with stress and peripheral inflammation promotes negative behavioral outcomes, such as anxiety, sickness, and cognitive decline. As illustrated in Figure 1, these data support the notion that monocyte trafficking to the brain is a key axis in immune-to-brain signaling that influences mood and behavior.

## Propagation and convergence of neuroimmune signaling is mediated by endothelial IL-1R1 expression

Related to previously discussed points, interactions at the neurovascular interface are critical for propagation of neuroinflammatory signaling related to RSD-induced monocyte trafficking to the brain. Classical neuroimmune communication studies showed that transduction of pro-inflammatory cytokines across the BBB and through circumventricular organs leads to microglia activation. Following activation by cytokines, microglia then produce secondary signals that can directly influence neuronal pathways (Quan and Banks, 2007). This is relevant in the context of RSD because peripherally-derived monocytes/macrophages potentiate neuroinflammatory signaling in proximity to the neurovascular interface. Indeed recent findings reveal that peripheral IL-1β is an important modulator of RSD-induced neuroinflammation and anxiety. For instance, development of primed myeloid cells, macrophage trafficking in the brain, and altered microglia morphology are prevented in IL-1R1^KO^ mice after RSD (Engler et al., 2008; Wohleb et al., 2011, 2014b). The lack of stress-associated neuroinflammation coincided with decreased anxiety-like behavior in IL-1R1^KO^ mice after RSD. Because microglia do not respond robustly to direct IL-1 stimulation (An et al., 2011), it is likely that another cellular intermediate such as vascular endothelial cells are necessary to propagate peripheral inflammatory signals. Thus, propagation of myeloid-derived signals into the brain requires complex signaling events at the neurovascular interface.

Recent evidence using novel transgenic tools with RSD revealed that endothelial IL-1R1 expression plays a critical role in the propagation and convergence of neuroimmune signaling. In these studies mice with selective knockdown of IL-1R1 on endothelial cells (eIL-1R1KD) (Li et al., 2012) had reduced neuroinflammatory gene expression and decreased anxiety-like behavior after RSD (Wohleb et al., 2014b). Contrary to findings with the ubiquitous IL-1R1^KO^ mice, eIL-1R1KD mice developed primed monocytes in circulation that successfully trafficked to the brain following RSD. These data indicate that peripheral myeloid cells recruited to the brain with RSD communicate with the endothelial cells using IL-1. Moreover, unlike IL-1R1^KO^ mice, eIL-1R1KD mice exhibited microglia activation following RSD (Wohleb et al., 2014b). Thus, IL-1 signaling was not necessary for stress-induced alterations in microglia morphology. Despite this, eIL-1R1KD reduced cytokine mRNA expression in microglia isolated from RSD-exposed mice. These data were interpreted to indicate that stress-induced neuroinflammatory signaling originated from both microglia and macrophages and was propagated by endothelial cells. Therefore, convergent signals from microglia, neurovascular endothelial cells, and monocytes are critical in immune-to-brain signaling that promotes anxiety-like behavior following RSD (Figure 4).

## Repeated stress exposure leads to neuroimmune sensitization and susceptibility to recurrent anxiety-like behavior

An important clinical component of stress research is the duration that stress-related behavioral adaptations persist. Findings from animal models revealed that microglia activation and trafficking of monocytes to the brain contribute to behavioral adaptations to stress (Wohleb et al., 2011, 2013; Hinwood et al., 2012; Kreisel et al., 2014; Sawada et al., 2014), but the role of neuroimmune signaling in long-term and recurrent stress-related behavioral disorders had not been addressed. For example, various chronic stressors cause neuronal atrophy and deficits in HPA activity that are implicated in long-lasting anxiety- or depressive-like behavioral changes (Vyas et al., 2003; Schmidt et al., 2007; Mizoguchi et al., 2008; Philbert et al., 2011), but the contribution of neuroimmune signaling was not considered. In fact, many of these neuronal and neuroendocrine alterations can be caused by pro-inflammatory cytokines and microglia activation. For example, pro-inflammatory transcriptional activity associated with microglia activation reduced neurogenesis in the hippocampus (Koo and Duman, 2008, 2009) and was implicated in reduced synaptic protein expression in the PFC (Kang et al., 2012). Thus, persistent neuroimmune sensitization may promote long-term behavioral and neurobiological adaptations to stress.

Similar to previously discussed reports, work in the RSD model revealed long-lasting changes in behavior, but in this case, brain monocytes and neuroimmune signaling had a significant role in the maintenance and re-occurrence of stress-induced anxiety-like behavior. For instance, mice exposed to RSD showed elevated pro-inflammatory cytokine expression in enriched microglia and increased macrophage populations in the brain that corresponded with prolonged anxiety-like behavior 8 days after stress cessation (Wohleb et al., 2014a). In addition, macrophages in the brain and microglia pro-inflammatory cytokine expression were no longer detected 24 days after stress, and this coincided with resolution of anxiety-like behavior at this 24 day time point. Despite the resolution of these parameters by 24 days, certain parameters were still persistently altered. This indicates that RSD caused priming or sensitization of neuroimmune responses. For example, enriched microglia showed increased expression of IL-6, CD14, and CX_3_CR1 that was associated with altered morphology 24 days after RSD. Moreover, examination of peripheral immune organs showed that RSD caused significant re-distribution of monocyte progenitors in the spleen that persisted for at least 24 days (Wohleb et al., 2014a).

Recent findings indicate that RSD caused prolonged neuroimmune alterations that persist after stress contribute to exaggerated neuroinflammatory responses following acute stress at later time points. Indeed, RSD-exposed mice remained sensitized to subsequent stress exposure 24 days after RSD (Wohleb et al., 2014a). For example, at this 24 day time point, re-exposure to a single cycle of social defeat caused the re-establishment of anxiety-like behavior and brain-monocyte trafficking. It should be noted that the single cycle of social defeat had no detectable effect on naïve mice, but caused the re-establishment of monocyte trafficking and anxiety in RSD-sensitized mice. Thus, one cycle of social defeat was considered to be a sub-threshold stressor. Moreover, because previous exposure to RSD caused mice to have an exaggerated response to a sub-threshold stress, RSD-exposed mice were termed to be “stress-sensitized” (Wohleb et al., 2014a). Collectively, these data showed strong kinetic relationships between the maintenance and recurrence of anxiety and brain monocyte trafficking. Furthermore, these kinetic relationships provide strong evidence that brain monocyte-trafficking re-established anxiety-like behavior in stress-sensitized mice.

**Figure 5:**
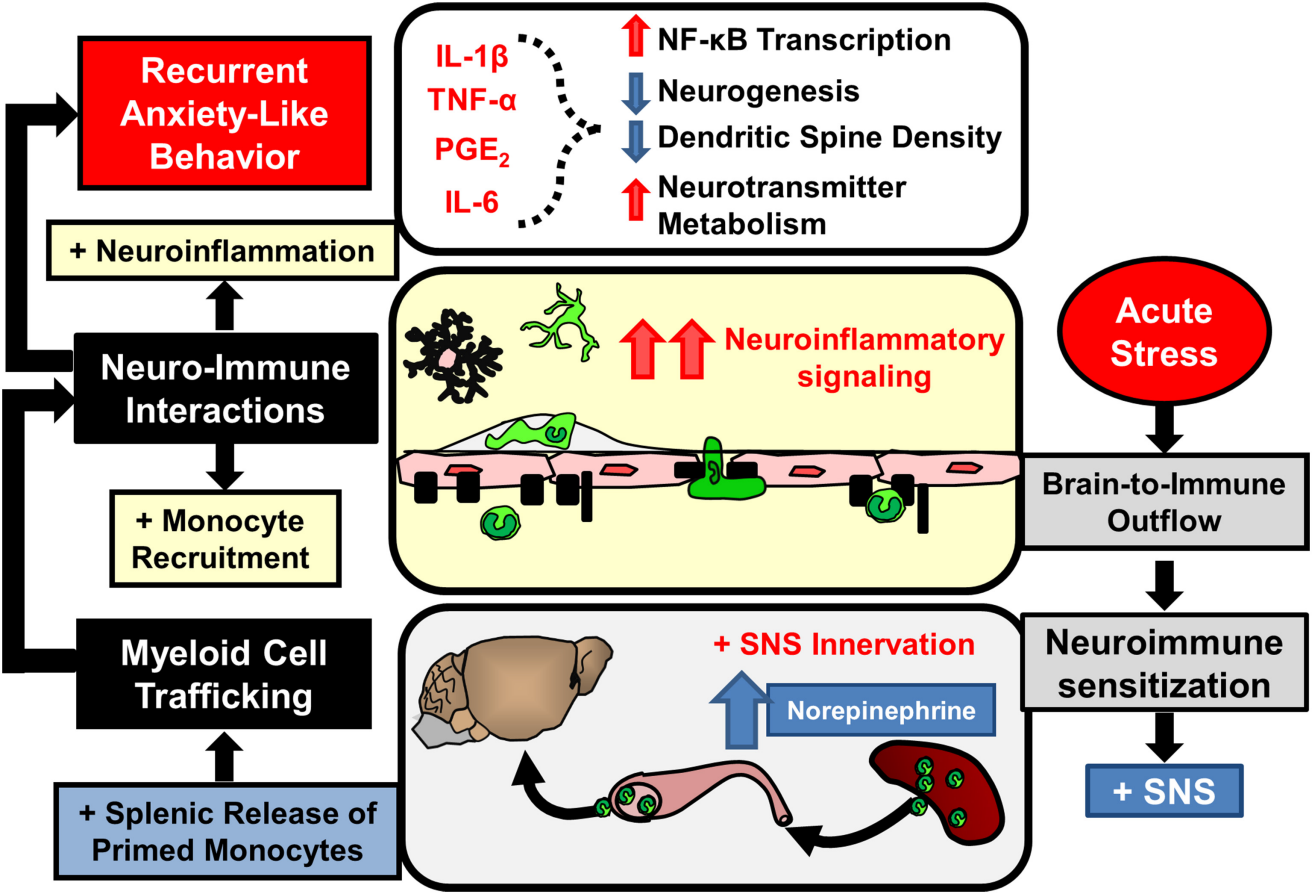
**Stress-induced neuroimmune sensitization and spleen-to-brain macrophage trafficking after acute stress leads to recurrent anxiety-like behavior**. Repeated stress exposure leads to neuroimmune sensitization and re-distribution of immature monocyte progenitors in the spleen. Following acute stress SNS activation leads to rapid release of monocytes that traffic to the brain and enhance neuroinflammatory signaling initiated by primed microglia. In these conditions elevated neuroinflammatory mediators (IL-1β, TNF-α, IL-6, prostaglandins) likely provoke recurrent anxiety-like behavior by reinforcing neurobiological alterations caused by prior stress exposure.

Notably, increased monocyte re-distribution following sub-threshold stress in sensitized mice was not associated with altered monocyte production or egress from the BM. In contrast, myeloid cell progenitors were increased in the spleen following RSD and sub-threshold stress caused release of monocytes from this splenic monocyte reservoir (Wohleb et al., 2014a). Other studies in models of myocardial infarction (Swirski et al., 2009), atherosclerosis (Dutta et al., 2012), and stroke (Seifert et al., 2012) revealed that the spleen acts as an important source of monocytes during inflammatory conditions. The role of spleen-to-brain monocyte trafficking in stress-sensitized mice was substantiated by the fact that splenectomy prevented brain monocyte trafficking and prevented re-establishment of anxiety-like behavior following sub-threshold stress (Wohleb et al., 2014a). As depicted in Figure 5, these results indicated that prior exposure to RSD caused primed monocytes to persist in the spleen for 24 days that subsequently trafficked to the brain and promoted the reoccurrence of anxiety following exposure to sub-threshold stress. In addition, pro-inflammatory cytokine levels in isolated microglia were augmented after sub-threshold stress in stress-sensitized mice and this response was attenuated in splenectomized mice. Thus, as illustrated in Figure 5, this study revealed that spleen-to-brain monocyte trafficking played a prominent role in the re-establishment of anxiety-like behavior and neuroinflammatory signaling in stress-sensitized mice (Wohleb et al., 2014a).

The mechanisms driving neuroimmune sensitization in the spleen following RSD have not yet been determined. Nevertheless, studies involving spleen-monocyte trafficking in other contexts provide some insight into these phenomena. For example, there are a couple of groups that have reported on the idea of spleen-monocyte trafficking in the context of cardiovascular disease and stroke (Swirski et al., 2009; Leuschner et al., 2012; Seifert et al., 2012). In particular, recent work revealed that spleen-monocyte trafficking was implicated in the acceleration of atherosclerosis in ApoE^−/−^ mice following myocardial infarction (Dutta et al., 2012). Myocardial infarction transiently increased the release of myeloid progenitors from the BM that seeded the spleen and contributed to extramedullary production of inflammatory monocytes that persistently trafficked to vascular lesions and promoted the progression of vascular lesions. This is a relevant finding because RSD also caused the release of immature and progenitor-like monocytes that seeded the spleen (Hanke et al., 2012; Wohleb et al., 2014a). In fact, there is evidence that RSD caused increased extramedullary monocytopoiesis in the spleen that persists in stress-sensitized mice. For example, increased CD11b^+^/Ly6C^+^/CD34^+^ monocytes were observed in the spleen following RSD that persisted in stress-sensitized mice for up to 24 days (Wohleb et al., 2014a). Thus, chronic induction of splenic monocytopoiesis may contribute to enhancement of spleen-monocyte trafficking in stress-sensitized mice.

There are several reports relating to the regulation of splenic monocyte egress that may be relevant to stress-sensitization. For example, following myocardial infarction, angiotensin II signaling contributes to the release of inflammatory Ly6C^hi^/CCR2^+^ monocytes that rapidly traffic to the lesion site and significantly contribute to pathology (Swirski et al., 2009). In these studies, deletion or inhibition of angiotensin II type 1a receptor prevented spleen monocyte trafficking and attenuated myocardial infarct volume. Moreover, angiotensin II administration independently caused monocyte egress from the spleen. This is relevant to stress-sensitization, because both acute and chronic stress increase angiotensin II in circulation (Jezova et al., 1998; Saavedra et al., 2005; Saavedra and Benicky, 2007). Similar to these reports, the role of spleen-to-brain monocyte trafficking in the exacerbation of stroke has also been reported on Seifert et al. (2012). In this report, cerebral artery occlusion caused monocyte trafficking from the spleen to the brain that augmented infarct size (Ajmo et al., 2008). For example, splenectomy prior to cerebral artery occlusion decreased infarct volume by about 80%. In these studies, egress of splenocytes is dependent upon peripheral noradrenergic signaling (Ajmo et al., 2009). This is particularly relevant because one cycle of RSD (i.e., sub-threshold stress) increased norepinephrine in both circulation and in the spleen (Hanke et al., 2012). Thus, these reports implicate noradrenergic and angiotensin II signaling in the release and traffikcing of splenic monocytes in stress-sensitized mice.

Based on these reports, norepinephrine and angiotensin II are key regulators of spleen monocyte trafficking, yet it is unclear why sub-threshold stress only caused splenic monocyte trafficking in stress-sensitized mice and not naïve mice. One possibility is that stress-sensitization alters the sensitivity of spleen monocytes to egress-related signals (e.g., angiotensin II or norepinephrine). In fact, there is evidence for this. Stress-sensitized mice exhibited increased monocyte progenitors in the spleen (Wohleb et al., 2014a), and in the context of cardiovascular disease, splenic monocyte progenitors have a higher propensity for egress and trafficking than the pool of monocytes available under homeostatic conditions (Dutta et al., 2012). An alternative possibility is that exaggerated monocyte trafficking in stress-sensitized mice occurs as a function of the magnitude of sympathetic response to the acute stress. Despite these possibilities, the underlying mechanisms of splenic sensitization following RSD are not fully understood.

One last point of discussion is the clinical relevance of RSD as a model of stress-sensitization and recurring anxiety. Although RSD-sensitization is not considered a model of posttraumatic stress disorder (PTSD), it can be argued that studying stress-sensitization within the RSD paradigm may have some basic relevance to this distinctly human condition. For instance, both RSD and PTSD involve a sensitizing event that is often both physical and psychological in nature (Bailey et al., 2004; American Psychiatric Association, 2013; Freeman et al., 2013). Similar to RSD, the sensitizing event or trauma predisposes the individual to recapitulate the behavioral and physiological responses following exposure to either generalized cues or subsequent stressful events (American Psychiatric Association, 2013; Wohleb et al., 2014a). Furthermore, both PTSD and RSD involve chronic maintenance of psychosocial deficits (American Psychiatric Association, 2013; Wohleb et al., 2014a). Despite these commonalities, PTSD remains a complex and distinctly human disorder that is unlikely to be fully modeled by RSD-sensitization in mice. However, mechanisms contributing to stress-sensitization following 6 cycles of RSD may be relevant to the biological events associated with PTSD in humans. In fact, it is increasingly evident that neuroimmune signaling contributes to the development and maintenance of PTSD and other chronic anxiety disorders. As reviewed by Pace and Heim (2012), there are strong clinical associations between recurring anxiety and inflammatory signaling, and these same associations are recapitulated in the RSD model. Taken together, evidence in the RSD-sensitization model, indicates that spleen-to-brain monocytes trafficking may be relevant to disorders involving recurrent anxiety.

## Summary

Exposure to chronic psychological stress promotes brain-to-immune and immune-to-brain communication that directly influences neurobiology and behavior (Figure 1). This review highlighted studies that show psychological stress promotes simultaneous activation of microglia and peripheral monocytes that directly influenced behavioral responses to stress. These studies revealed that noradrenergic signaling in the brain contributed to microglia activation within threat appraisal regions (Figure 2). Next, sympathetic outflow to the periphery enhanced the production and release of inflammatory monocytes from the bone marrow that trafficked throughout the body (Figure 3). Furthermore, we reviewed studies that show both stress and peripheral inflammation promote the accumulation of monocytes and macrophages in the brain. These studies revealed that brain monocyte trafficking involves dynamic interactions between cells of the neurovascular unit. These cells produce cytokines, chemokines, and cell adhesion molecules that facilitate accumulation of macrophages in the brain (Figure 4). Signals from BM-derived macrophages and activated microglia converge within the brain to promote neuroinflammatory signaling leading to the development of prolonged anxiety-like behavior (Figure 4). Moreover, this review covered recent evidence that spleen-to-brain monocyte trafficking contributes to stress-sensitization and recurring anxiety (Figure 5). Taken together, these studies reveal a novel axis of immune-to-brain communication involving monocytes trafficking to the brain that influences mood and behavior.

### Conflict of interest statement

The authors declare that the research was conducted in the absence of any commercial or financial relationships that could be construed as a potential conflict of interest.
